# Estimation of GHGs emission from traditional kilns charcoal production in northwestern Ethiopia: Implications on climate change

**DOI:** 10.1016/j.heliyon.2024.e41015

**Published:** 2024-12-07

**Authors:** Biruk Belay, Dawit Diriba, Feyera Senbeta

**Affiliations:** a Department of Economics, College of Business and Economics, Aksum University, Aksum, Ethiopia, P.O.Box 1010, Aksum, Ethiopia; b Center of Environment and Development, College of Development Studies, Addis Ababa University, Addis Ababa, P.O.Box 1176, Addis Ababa, Ethiopia

**Keywords:** Traditional charcoal production, Greenhouse gas emissions, Global warming, Climate change, Mitigation measures, Ethiopia

## Abstract

Rural areas in Ethiopia serve as the primary source of charcoal for urban populations, mainly produced using traditional kilns. However, this traditional method significantly contributes to greenhouse gas (GHG) emissions, exacerbating climate change and deforestation. While banning charcoal production is not currently feasible in Ethiopia because of the lack of affordable alternative energy sources (fuel), improving the efficiency of the traditional production system can mitigate the climate impact caused by charcoal production. This study assessed GHG emissions from traditional charcoal production in Awi zone, northwestern Ethiopia, using primary data from 18 sample kilns and secondary data from literature values. Employing a carbon balance approach, we estimated that, on average, 63 % of the original wood carbon was lost as gaseous products, resulting in 1671 g of carbon released per kg of charcoal produced in Awi zone. Our results also indicate that the average primary global warming impact (PGWI) for the 18 sample kilns was found to be 7.6 kg CO2-eq per kg of charcoal produced. Within this, the less efficient kiln production system, constituting 6 out of the sample, contributed 1.5 times more to global warming (9.43 kg CO_2_-eq per kg of charcoal produced) compared to the more efficient kiln system (6.25 kg CO_2_-eq per kg of charcoal produced for the same number of kilns). The policy implication of our finding is that any interventions aiming at mitigating climate change through reduction of GHG emissions from charcoal production must focus on improving the conversion efficiency of the traditional kiln currently used in addition to promoting the use of sustainably harvested wood.

## Highlights

• Traditional kilns are predominantly used for charcoal production in Ethiopia.
• It is characterized by a high rate of greenhouse gas emissions that exacerbate climate change and deforestation.
• Banning charcoal production is not currently feasible in Ethiopia because of the lack of affordable alternative fuels.
• Improving the efficiency of the traditional production can mitigate the climate impact of it.

## 1 Introduction

Climate change mitigation, energy security and poverty reduction have recently been the most important aspect of sustainable energy development policies for global communities [1]. Despite its environmental consequences, charcoal production provides energy security and livelihood in many developing countries; moreover, charcoal production has the potential to mitigate climate change as the efficiency of the kilns used for charcoal production in these countries are typically below the technological potential, thus efficiency improvement has substantial potential to reduce greenhouse gas (GHG) emissions [2]. In Ethiopia, 95 % of the population still relies on biomass fuels, with firewood (82 %) and charcoal (8 %) most used followed by dung and crop residues (5 %) [3]. In 2015, 66 % of the total wood fuel (firewood and charcoal) consumed in the country was unsustainably sourced from natural high forests and woodlands [1,4]. According to FAOSTAT, in 2022 the country produced 4.95 million tons (Mt) of charcoal, representing 8.65 % of global production and placing Ethiopia 2nd in the world, next to Brazil [5]. In Ethiopia nearly all the charcoal is produced using traditional earth mound kilns in rural areas and is consumed in urban areas [1]. Owing to population growth and urbanization, the demand for charcoal fuel has grown in the recent years, and is expected to continue growing in the near future due to lack of affordable alternative energy sources [2,6].

Charcoal is a carbon rich fuel produced by heating wood in a slow pyrolysis process, in limited oxygen, called carbonization [7]. This process converts wood products to charcoal fuel and other multiple by-products including solids (ash and brands [i.e., partially carbonized wood products]), liquids (condensables) and gaseous products such as GHGs. While there are other contributors to global warming, such as changes in land use and natural processes, the overwhelming scientific consensus is that emissions of GHGs from charcoal production and other human activities such as burning of fossil fuels and non-fossilized vegetative or organic fuels are the dominant driver of global anthropogenic emissions and climate change [8]. They have contributed more than 76 % of the globally increased greenhouse effects [9]. Climate change has recently caused huge worldwide concern that initiated the signing of several international agreements aiming to mitigate its impact by reducing GHG emissions [10].

Despite Ethiopia contributing less than 0.5 % of global GHG emissions in 2018, the government issued a series of policy measures that includes the Climate Resilient Green Economy Strategy (CRGE) in 2011, National Adaptation Program (NAP) in 2020 and National Determined Contributions (NDC) in 2015 and 2021 to mitigate climate change by reducing GHG emissions [[11], [12], [13]]. These strategies specified the sources of GHG emissions and proposed mitigating activities to reduce GHG emissions [1,12,13]. The proposed activities in CRGE include improving crop and livestock production practices while reducing emissions, protecting and restoring forests, expanding electricity generation from renewable energy, and adopting modern, energy-efficient technologies [10]. In the updated NDC sector-specific priority actions for mitigation and adaptation strategies were identified and prioritized such as undertaking large-scale investments in the potential of hydro, solar, and wind energy as well as undertaking green legacy initiatives and afforestation projects. Ethiopia's commitment to reduce GHG emissions was expressed in the updated which predicted a 68 % reduction in GHG emissions from the conventional development practices by the year 2030 [12,13].

Despite these efforts, the Ethiopian government didn't specify the potential contribution of the currently expanding charcoal production sector to GHG emissions nor did they identify the improvement measures necessary to reduce GHG emissions from this sector [[2], [12], [14]]. A knowledge gap exists regarding the potential contributions of the charcoal sector to climate change mitigation [1,2]. Furthermore, the absence of empirical information on GHG emissions from charcoal production hinders understanding of the potential benefits of improving the charcoal production system to the environment (climate change mitigation), energy security and livelihoods in Ethiopia, and requires interventions in this sector. However, in order to carry out any intervention plans to reduce GHG emissions from charcoal production, estimating GHG emissions from charcoal production is the first step to make.

The purpose of this study was to quantify GHG emissions from traditional charcoal production processes in Awi Zone of Ethiopia. Specifically, this study aimed to estimate the emission factors for the traditional earth mound charcoal production technology in the study area and compare them with previous findings for the same technology. The objective also included estimating the total GHG emissions and GWI of the charcoal production system in the study area. Ultimately, the study aimed to provide policy recommendations for the development of climate-friendly sustainable charcoal production sector in the country.

## 2 Methodology of the study

### 2.1 Description of the study area

**Fig. 1:**
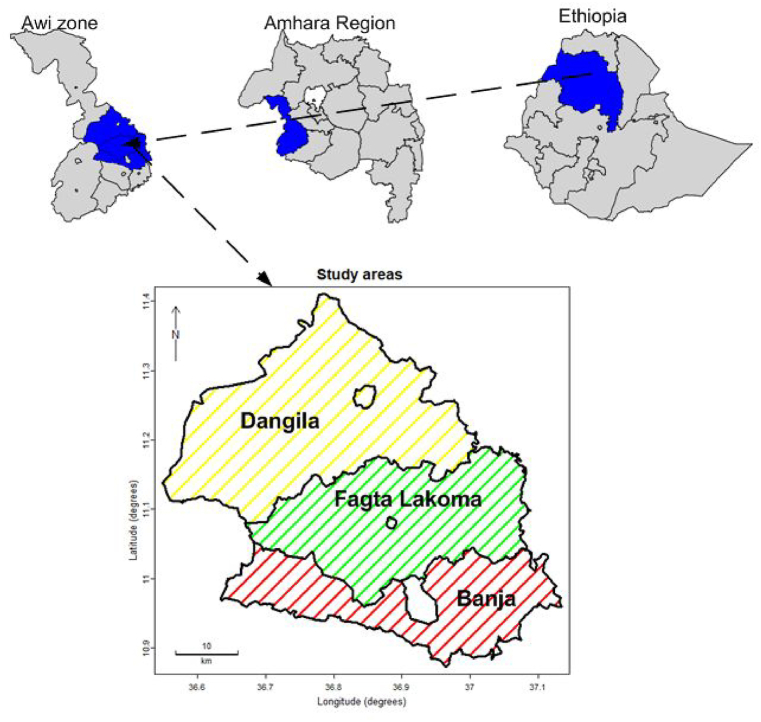
Location of the study districts adopted from Ethiopian Mapping Agency.

This study was conducted in Awi Zone which is found in the Amhara Regional State, northwestern Ethiopia (Fig. 1). Awi Zone is situated between 10°23′N and 10°85′N latitudes and 36°35′E and 36°57′E longitudes with an altitude ranging between 1800 and 3100 m above sea level. Awi Zone is considered as a study area based on the following reasons. First, Awi Zone has more private plantation forest than other Zones in the country [15]. For example, approximately 67 % of Fagtia Lekoma, one of the districts in Awi zone, is covered by plantation forest [15]. Furthermore, unlike other parts of the country, Awi Zone relies on private plantation for wood inputs, with nearly all plantation forests utilized for charcoal production [1,4]. Second, charcoal production is the major source of income widely practiced among many households in the zone. For instance, in Fagita Lekoma district, nearly 90 % of the population is directly or indirectly involved in activities related to *A. decurrens* plantations for charcoal production [16]. Third, much of the charcoal supply for major urban areas including Addis Ababa comes from this zone. According to Ref. [17], among the five charcoal supply inlets to Addis Ababa, Awi Zone was the 2nd largest producer and supply inlet (28.8 % of the total charcoal supplied to the city) next to Kalliti inlet. The supply of charcoal from this zone reached even as far as Eritrea during the brief border opening in 2018 after the peace deal between the two countries. Finally, plantation of *Acacia decurrens,* which serves as an important income source through charcoal production, is highly abundant in this zone [15]. According to the Center for International Forestry Research's (CIFOR) [18], smallholder plantations practices of *A. decurrens* in Awi Zone were identified as effective for expanding forest management practices elsewhere in the country.

### 2.2 System boundary

**Fig. 2:**
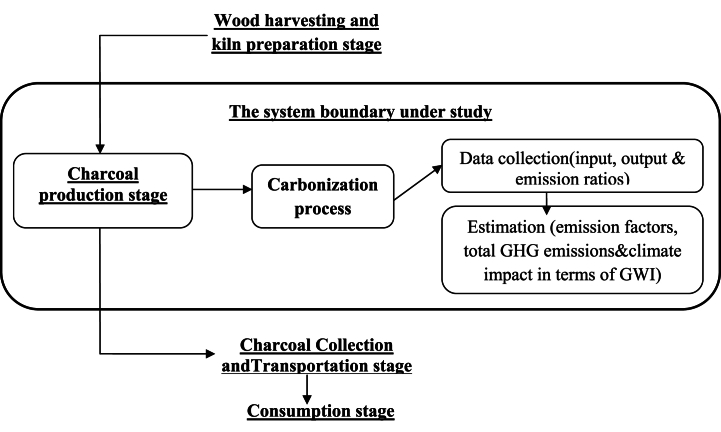
The life cycle of charcoal fuel and the system boundary under analysis.

To quantify the GWI of the traditional charcoal production system, this study considered only the production stage of the charcoal life cycle. This limitation was attributed to the lack of information regarding the entire life cycle of the charcoal fuel. The contribution of the production stage of the charcoal product to GHG emissions ranges between 53 and 82 % of the total GHG emissions in the entire charcoal fuel life cycle [2,17]. Therefore, the charcoal production stage is a greater contributor to global warming than the other stages of the value chain combined [[18], [19], [20], [21], [22], [23], [24]]. Fig. 2 below indicates the life cycle of charcoal fuel and the system boundary that was taken into account for the estimation of GHG emissions in this study. The GHGs included in the study were the two gases listed in the Kyoto Protocol, CO2 and CH4. In the case of complete combustion of wood with full access to oxygen, the wood burns completely, producing only CO2 and water (H2O). However, when wood undergoes partial combustion with limited access to oxygen, charcoal is produced along with CO2 and H2O. This process also produces additional products called products of incomplete combustion (PIC) such as carbon monoxide (CO), methane (CH_4_), total nonmethane hydrocarbons (TNMHC), and total suspended particulates (TSP) [23,24]. The emission of PIC is quite important because CH4, TNMHC, and CO have higher global warming potentials (GWP)-or ability to cause warming of the Earth's atmosphere per kilogram of carbon-than CO2 [24]. Among the PIC, CH4 is a GHG. While this study has placed more emphasizes on GHG emissions (CO2 and CH4) because of their direct impact on the climate, it also reports the results of CO and TNMHC, as they indirectly affect global warming through atmospheric photochemical reactions that, in turn, influence GHG levels [23,24].

### 2.3 Sampling, data sources and methods of collection

#### 2.3.1 Sampling techniques

**Fig. 3:**
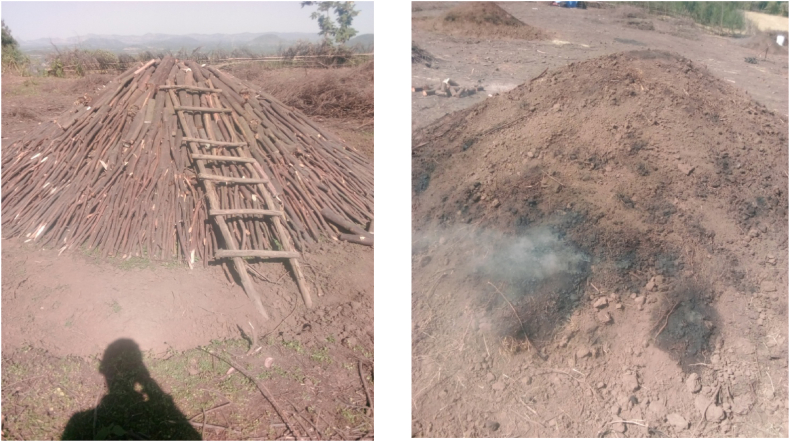
**(a)** Building traditional earth mound kilns in the study area **and (b)** Carbonization process using traditional earth mound kilns in the study area. Sources: Filed survey, 2020.

A multistage sampling procedure was used to select sample kilns in consultation with district officials, charcoal traders and producers. Initially, 3 districts (Fagita Lekoma, Banja and Dangela) from Awi zone were chosen, followed by the purposive selection of 9 study villages (3 from each district). These villages were chosen based on discussion with zonal experts, district officers, and local producers, focusing on significant charcoal production on a regular basis. Finally, 18 charcoal-producing households who were planning to produce charcoal at the time of data collection were randomly selected for the study. All selected sample kilns were traditional earth-mound kilns, see Fig. 3 below. While the charcoal production system in Ethiopia, specifically the wood sources for charcoal production, varies from place to place, their main characteristics are shared throughout the country [1]. Moreover, according to extensive evidence, nearly all charcoal in Sub-Saharan Africa (SSA) is produced in traditional earth-mound kilns [25]. Consequently, the 18 sample kilns that were considered in this study are representatives of the charcoal production practice not only in Ethiopia but also in SSA.

#### 2.3.2 Data types, sources and collection techniques

Since the purpose of the sampling was to estimate total GHG emissions from charcoal production, knowledge of all the quantities of inputs-outputs and the emission factors of the corresponding gases are required [21,22]. Thus, the data are classified into input-output data and emission data. In this study, both primary and secondary data sources were used to collect these data. The two types of data, their method of collection and sources are discussed in detail below.

##### 2.3.2.1 Input-output data

The amount of biomass input used and outputs produced were collected by conducting field measurements on the 18 sample kilns from January to March 2020 with the assistance of charcoal producers. Additional data on the carbon contents of the wood and charcoal were obtained from other studies.

The field surveys comprised four steps. Initially, the corresponding author, accompanied by three field research assistants, visited the 18 sample charcoal producers and their kiln sites. This aimed to identify the tree species planned for the kiln and gather estimated dates for tree cutting. In the second step, the research team revisited the production sites to observe tree felling, count felled trees before cross-cutting, and weigh cross-cut trees using a 100 kg balance. Charcoal producers used axes for tree felling and cutting; cross-cut trees were left outdoors for several days to dry. After drying, the third step involved re-weighed the air-dried wood using a 100 kg balance just before inclusion in the kiln for carbonization. Additionally, the height and diameter of the dried wood were measured using a measuring tape, as these parameters impact carbonization efficiency. Measurements of the 18 sample charcoal kilns’ sizes were taken using a measuring tape. In the final step, the research team revisited the field to weigh the charcoal and brands from the kilns. After carbonization was complete, brands and charcoal were separated, with the charcoal filled in 50 kg labeled sacks for marketing. The number of sacks produced by each kiln was counted and weighed using a 100 kg balance. Brands, used as a cooking fuel by producers or left on the kiln site with ashes, were weighed with a 100 kg balance.

The efficiency of the charcoal production is typically given as ratio of charcoal output to dry weight of wood input [25]. Even, air-dried wood still has a moisture content of 10–20 %, which means the remaining moisture must be accounted for when calculating kiln conversion efficiency. This is because carbonization (an exothermic reaction process) only begins once the wood is completely dry [27]. The exothermic process takes heat energy from burning wood to evaporate the water; leaving less wood available for conversion to charcoal during the carbonization phase [28]. Furthermore, if yield is measured on a wet or air-dry (rather than oven-dry) basis-i.e., the weight of charcoal output divided by the wet weight of wood input-the moisture in the wood increases the denominator, which decreases the apparent yield [28]. To avoid complications from differing moisture contents, wood should be measured on an oven-dry basis [29]. Properly measured conversion efficiencies allow for comparison between different charcoal-making methods.

Thus, in this study the charcoal recovery percentage was calculated on a dry mass basis, after determining the oven-dry weight and moisture content of the wood. To obtain oven-dry weights, we first determined the moisture content (MC) of a representative sample of wet wood from each kiln. The samples were put in an oven at 105^0^C and periodically weighed until no further weight loss occurred. The final weight was recorded as the sample's oven-dry weight. The moisture content was then calculated on an oven-dry basis [(sample wet wood weight – sample oven dry wood weight)/sample oven dry wood weight] following the method by Smith et al. [23]. Finally, the oven-dry weight of wood for each kiln was determined (wet wood weight/(1 + MC)).

The carbon (C) content in the input-output parameters (wood, charcoal, brand, ash and condesables) is crucial for estimating the C balance of emitted gases. Since these parameters are difficult to determine in the field, literature values were used. In this study, the kilns were charged with Acacia or Eucalyptus tree species. Wondie et al. [30] reported a carbon content of 47 % for Acacia species in Awi zone, whereas Smith et al. [23] reported a carbon content of 44 % for Eucalyptus species in Thailand. Thus we assume average carbon content of 45.5 % of the total biomass, to estimate the carbon content in the wood input. Our assumption is within the range of the C content in wood recommended by the CDM's consolidated GHG database for the informal charcoal sector (45 %) and assumptions made by various studies (44–48 %) [27,[31], [32], [33]]. Similarly, literature values were used to determine the C content of charcoal and by-products (brand, condensable and ash). Smith et al. [23] reported an average C content of 74.75 % for charcoal produced from Eucalyptus species in Thailand and Pennise et al. [24] reported 74.80 % for charcoal produced from Acacia species in Kenya. Therefore, the average C content of 74.78 % from these two studies was used for charcoal produced in the study area and this is close to the value assumed by Quantis [27] (73.3 %). A brand C content of 52.17 % of the brands mass was used in this study averaged from a C content of 52.33 % reported by Smith et al. [23] and 52.00 % reported by Pennise et al. [24]. Finally, condensable and ash are a vital component of the carbon balance calculation in the case of charcoal production [19,23,24,27], and in this study, because we did not measure condensable and ash of the production process, the carbon content of these products were estimated based on literature values. The carbon contents in condensables and ashes, as measured by Smith et al. [23] and Pennise et al. [24], are presented in kg C per kg of charcoal produced. According to the average values from Smith et al. [23] and Pennise et al. [24], 0.047 kg of C is produced in the condensables and 0.0021 kg of C in the ash per kg of charcoal (see Table 2 below). These values are also used by the CDM's consolidated GHG database for the informal charcoal sector and by Quantis [27].

##### 2.3.2.2 Emissions data

Emission data, specifically, emission ratios for CO, CH4, TNMHC and TSP with respect to CO2 were obtained from literature values reported by Smith et al. [23] and Pennise et al. [24]. These studies monitored emissions from traditional charcoal production processes, representing the most comprehensive monitoring studies available in the field. Moreover, the Clean Development Mechanism (CDM) ‘‘*consolidated GHG database for the informal charcoal sector”* was used to validate data collected from Smith et al. [23] and Pennise et al. [24]. The CDM database is the most complete GHG database for the traditional charcoal production system, considering both quantitative (number of test studies included) and qualitative (quality of the validation processes) perspectives. Similarly, Bailis et al. [19] estimated pollutants emissions using emissions ratios obtained from Brocard et al. [34].

It is important to mention here that conducting direct measurements of emissions from the charcoal production process would have been highly useful. However, the challenges associated with field measurements in the traditional production system, along with financial and time constraints, prevented such measurements in this study. Despite drawbacks, a review of literature and databases emerged as the simplest and most feasible method for obtaining required data, considering the encountered problems and weaknesses in not adopting a direct measurement of emissions. A Summary of the data types, collection method and sources is presented in Table 1 below.

**Table 1:** Key parameters (data types), methods of collection and sources.

Data type (Parameters)	Method of collection	Source
Input		
• Quantity of biomass used for charcoal production	• Field measurement of the amount of wood used	• Study site
• Moisture contents of the biomass	• Calculated from field measurement	• Study site
• C content of the biomass used	• Derived from field measurement and literature	• Study site & Literatures
**Process**		
• Kiln size	• Field measurement of the size of kilns used	• Study site
• Kiln efficiency	• Calculated from field measurement	• Study site
**Output**		
• Quantity of produced charcoal	• Field measurement of the amount of charcoal produced	• Study site
• Carbon C of produced charcoal	• Derived from field measurement and literature	• Study site & Literatures
• Mass & C content of by-products	• Derived from field measurement and literature	• Study site & Literatures
**Emission ratios**		
• CO/CO_2_	• Derived from literature	• Literatures
• CH_4_/CO_2_	• Derived from literature	• Literatures
• TNMHC/CO_2_	• Derived from literature	• Literatures
• TSP/CO_2_	• Derived from literature	• Literature

**Table 2:** Literature values used to estimate GHG emissions.

Parameters	Source		Average values used for the current study
	Smith et al. [23]	Pennise et al. [24]	
Carbon content of input-output products			
C% mass in wood	44.00 %	44.00 %	45.50 %^a^

C% mass in charcoal	74.75 %	74.80 %	74.78 %
C% mass in brands	52.33 %	52.00 %	52.17 %
C mass in Condensables (kgC/kg charcoal)	0.045	0.048	0.047
C mass in Ash (kgC/kg charcoal)	0.0035	0.00064	0.0021
Emission ratios			
CO/CO_2_	0.3021	0.2051	0.2536
CH_4_/CO_2_	0.0655	0.0790	0.0722
TNMHC/CO_2_	0.2234	0.1438	0.1836
TSP/CO_2_	0.0029	0.0269	0.0149

a This average was computed by including a carbon content of 47 % for acacia decurrence tree reported in the study area by Wondie et al. [30].

### 2.4 Quantifying the GWI of charcoal production

The GWI for the charcoal production system in the study area was estimated by calculating the total GHG emissions from charcoal production in the study area using an indirect approach that combined information from the field survey and assumed values obtained from previous similar studies.

#### 2.4.1 Quantification of GHG emissions from charcoal production process

The two variables that affect the total amount of emissions of a specific gas from charcoal production are the quantity of charcoal produced and the emission factor of that specific gas. In this study the amount of charcoal produced were determined from field measurements, whereas the emission factors (EFs) of charcoal production for gaseous products were determined based on carbon balance approach following the same procedure used by Bailis et al. [19], Smith et al. [23], Pennise et al. [24] and Lacaux et al. [31]. The carbon balance approach is based on the assumption that the original carbon (C) contained in the wood input used for charcoal production is equal to the sum of the C contained in the solids (charcoal, brands, and ash), liquid (condensable) and gaseous output (CO_2_, CO, CH_4_, TNMHC, TSP) after the carbonization process [19,23,24]. Mathematically, the C balance between the initial mass of C in the wood input and the amount of C diverted into the different products of the charcoal production process can be expressed as shown in Eq. (1):

(1) $$C_{wood}=C_{charcoal}+C_{brand}+C_{ash}+C_{condensables}+C_{{CO}_{2}\;}+C_{CO}+C_{{CH}_{4}\;}+C_{TNMHC}+C_{TSP}$$

Dividing both sides of Eq. (1) by $C_{{CO}_{2}\;}$ and rearranging it gives a series of emissions ratios (ERs) with respect to CO_2_ [17,21,22] as shown in Eq. (2):

(2) $$\frac{\left(C_{wood}–C_{charcoal}-C_{brand}-C_{ash}-C_{condensables}\right)}{C_{{CO}_{2}\;}}–\frac{\left(C_{CO}+C_{{CH}_{4}\;}+C_{TNMHC}+C_{TSP}\right)}{C_{{CO}_{2}\;}}=1$$

$$\text{Define}\;\left(C_{CO}+C_{{CH}_{4}\;}+C_{TNMHC}+C_{TSP}\right)/C_{{CO}_{2}\;}=K$$

Calculation of this ratio (K) necessitates knowledge of the C content of the wood input used and the produced outputs, parameters challenging to determine in the field [32]. However, in this study, the kilns were charged with a tree species with known average C content of 45.5 % for Acacia and Eucalyptus tree [21,28]. We also assumed an average value of C contained in charcoal, brands, ashes and condensables from Smith et al. [23] and Pennise et al. [24] (see Table 2 above). Once the C contained in the wood, charcoal, brand, ash and condensables are known, the next step is calculating the unknown amount of C emitted as CO_2_, CO, CH4, TNMHC and TSP from charcoal production. First, solving Eq. (2) for $C_{{CO}_{2}\;}$ provides the amount of C emitted as CO_2_ from charcoal production as expressed in Eq. (3):

(3) $$C_{{CO}_{2}\;}=\frac{\left(C_{wood}–C_{charcoal}-C_{brand}-C_{ash}-C_{condensables}\right)}{\left(1+K\right)}$$

Then, the total amounts of C emitted in the form of other gases (CO, CH_4_, TNMHC or TSP) were calculated by multiplying $C_{{CO}_{2}\;}$ by their ERs relative to CO_2_ as shown in Eq. (4):

(4) $$C_{i}={ER}_{\left(i/{CO}_{2}\right)\;}\times C_{{CO}_{2}\;},for\;i=CO,{CH}_{4},TNMHC,or\;TSP$$

Typically, the emission information for an activity, such as charcoal production, is represented by the quantity of a particular gas emitted per amount of charcoal produced, which is known as emission factor [32]. Emission factor (EF) can be expressed either on a Carbon (grams (g) of C gas emitted per kilogram (kg) of charcoal produced) or on a mass basis (g of gas emitted per kg of charcoal produced). The Emission factors (EFs) for each gas on a carbon basis (g of C emitted as gas h (CO_2_, CO, CH_4_, TNMHC, or TSP) per kg of charcoal produced) can be expressed by Eq. (5):

(5) $${EF}_{h}\left(C\right)=\frac{C_{h}}{charcoal\;\left(kg\right)}\times 1000$$

where ${EF}_{h}\;\left(C\right)$ is the EFs of gas h on carbon basis (g C per kg charcoal produced) and $C_{h}$ is kg of carbon emitted as gas h (CO_2_, CO, CH_4_, TNMHC, or TSP). Following the same procedure as Bailis et al. [19],the EFs for each gas on mass basis were calculated by multiplying the ${EF}_{h}\;\left(C\right)$ by the molecular ratio of gas h relative to C as expressed in Eq. (6):

(6) $${EF}_{h}\left(m\right)={EF}_{h}\left(C\right)\times {MR}_{\left(h/C\right)\;}$$

where ${EF}_{h}\left(m\right)$ is the EFs of gas h on mass basis (g h per kg charcoal produced) and ${MR}_{\left(h/C\right)\;}$ is the ratio of the molecular weights of gas h to C. In this study we employed EFs of the gases on mass basis to characterize emissions from charcoal production.

Thus, the total amounts of emissions of gas h from charcoal production in the study area were calculated by Eq. (7).

(7) $${TE}_{h}={Q_{charcoal}\times EF}_{charcoal,h}\text{,}$$

where ${TE}_{h}$ is the total emissions of gas h in kg, $Q_{charcoal}$ is the quantity of charcoal produced in kg and ${EF}_{charcoal,h}$ is the EF of gas h in g gas h emitted per kg charcoal produced.

#### 2.4.2 Calculation of GWI of charcoal production system in the study area

The GWI of charcoal production in terms of CO_2_ equivalent (CO_2_-eq) for the study area were calculated by adding the product of the total emissions of the gases (CO_2_, CO, CH_4_, and TNMHC) and their global warming potential (GWP) using Eq. (8):

(8) $$GWI=\sum_{j}\left({TE}_{j}\times {GWP}_{j}\right),for\;j={CO}_{2},CO,{CH}_{4},and\;TNMHC$$

where, ${TE}_{j}$ is total emissions of gas j, and ${GWP}_{j}$ is the GWP of gas j used to express the contribution of gas j to global warming relative to CO_2_ (ton CO_2_-eq/ton gas *j*). The concept of GWP is a relative measure that shows the amount of heat a GHG traps in the atmosphere compared to an equivalent amount of CO_2_, consequently the GWP of CO_2_ is 1 [27,35,36].

This study estimated the primary GWI and the total GWI following Smith et al. [23] and Pennise et al. [24], primary GWI (PGWI) includes only GHGs (i.e., CO_2_ and CH_4_) and the total GWI (TGWI) includes all the four gases including CO and TNMHC [21,22]. Moreover, in the study area, almost all the charcoal were produced from sustainably sourced wood from plantation trees. Consequently, PGWI and TGWI were calculated by excluding biogenic CO2 emissions, assuming the entire CO_2_ is recycled. This assumption is based on the understanding that CO2 released during the production process is equivalent to the CO2 removed from the atmosphere through photosynthesis during the growth of the trees used for charcoal production. Therefore, no biogenic CO2 emissions are accounted for in the production process, in accordance with IPCC guidelines [37]. Thus, primary global warming impact (PGWI) was calculated by including only CH_4_ using Eq. (9):

(9) $$PGWI={TE}_{{CH}_{4}}\times {GWP}_{{CH}_{4}\;}$$

Whereas, the TGWI was calculated by including CO and TNMHC in addition to CH_4_ using Eq. (10):

(10) $$TGWI=\sum_{non-{CO}_{2}}\left({TE}_{non-{CO}_{2}}\times {GWP}_{non-{CO}_{2}}\right),for\;non-{CO}_{2}={CH}_{4},CO,TNMHC$$

The GWP of the $non-{CO}_{2}$ gases in CO_2_-eq are dependent on the time period over which the equivalency is calculated since different gases have different residence periods in the atmosphere [36]. The time horizons of 20 and 100 years are the most widely used time horizons. Table A2 in the appendix shows the conversion factors for a gram of $non-{CO}_{2}$ emissions expressed as gram of CO_2_-eq.

For a more comprehensive evaluation of the possible benefits of the charcoal production system in the study area and to show the full impacts when all the wood used sourced unsustainably, the analysis presented the emissions including biogenic CO2. Finally, the study further assessed the impact of improving the conversion efficiency on reducing wood usage and GHG emissions during charcoal production by using three different hypothetical levels of improved kiln efficiency compared to the average conversion efficiency in the study area.

## 3 Results and discussion

### 3.1 Measurement results in mass and carbon basis

To quantify GHG emissions and GWI of charcoal production, information about the mass and carbon content of the inputs and outputs involved in the charcoal production process is required. Thus in this section the measurement results of biomass and carbon flows of the charcoal production process in the study area are presented.

#### 3.1.1 Input-output measurements and kiln characteristics

Table 3 presents the average values of kilns size, weight of wet, air-dried and oven-dry wood used, moisture content of the wood, weight of charcoal produced and conversion efficiency of the kilns for each of the 3 districts.

**Table 3:** Average values of kiln size, wood, charcoal, and conversion efficiency by district.

District	No. of Kiln	Kiln Vol. (m3)	Wet wood cut (kg)	Air dried wood (kg)	MC air dry basis (%)	MC oven dry basis (%)	Oven dry wood (kg)	Charcoal weight (kg)	Effi. on oven-dry basis (%)
Banja	6	5.33	3095.50	2437.00	26.94	35.87	2276.83	416.00	18.32
Fagita	6	8.50	5245.83	4148.00	26.91	35.88	3874.17	580.83	15.16
Dangila	6	4.67	2530.17	1981.33	27.38	36.32	1851.50	358.33	19.97

Ave.		6.17	3623.83	2855.44	27.08	36.02	2667.50	451.72	17.82

Charcoal producers cut on average around 3624 kg of wood for a single kiln (Table 3). Harvested wood is air-dried for about an average of 16 days before its loaded into the kiln for charcoaling. After two weeks of drying, the average weight of the air-dry wood was about 2855 kg, while the average weight of the oven-dry wood was about 2668 kg (Table 3). The average moisture content (MC) of the wood on oven-dry basis varies little from district to district with a study area average of about 36 % (Table 3). An average of around 452 kg of charcoal were produced per kiln in the study area with the highest charcoal producing district in descending order were in Fagita (580.83 kg), Banja (416.00 kg), and Dangila (358.33 kg)-with an average conversion efficiency of 17.82 % on oven-dry basis (charcoal weight/oven-dry wood weight) (based on measurement presented in Table A3 in the appendix). The average kiln efficiency varies from the lowest in Fagita district (15.16 %) to the highest in Dangila district (19.97 %). The average kiln conversion efficiency rate for the present study was within the range of previous study reports for the traditional earth mound [[38], [39], [40], [41], [42], [43], [44], [45]]. These studies provided charcoal kiln efficiency values ranging from 10 to 25 %. Moreover, the results of the efficiency of the kilns found in the study area validate the assumed value used by UNEP for the assessment of the sustainability of the charcoal sector in Ethiopia. In the assessment UNEP assumed a kiln efficiency value of 17 % which falls within the range of the kiln efficiency found in this study [1].

The result of the field observation presented in the appendix (Table A3) shows that the 18 sample kilns use trees from two species including *Acacia Decurens* (50 %) and *Eucalyptus globulus* (28 %) and a mix of these two species (22 %). The major sources are smallholder plantation trees in the study area. Households in Awi zone are allowed to produce charcoal only from a plantation forest either from their own plantation or purchased from others plantation and it is mandatory to get permission to produce charcoal from local officials. The formal and informal communications with local officials and the field observations of this study confirmed that the sources of the wood for charcoal making were mainly from plantation forests in the study area.

#### 3.1.2 Carbon flows

The carbon content of input-output products (wood, charcoal, brand, condensable and ash) for each of the 18 charcoal kilns were calculated based on the above result and literature values (Table 2 above).

Table 4 presents C content of the wood used for charcoal production in the study area-varies from the lowest in Dangila district (842.47 kg C) to the highest in Fagita district (1762.83 kg C), with zonal average value of about 1214 kg C. Moreover, this study's results shows that the average amount of carbon stored in the produced charcoal was around 338 kg C. The average carbon stored in charcoal shows variation among Dangila, Banja and Fagita districts (Table 4). The wide variation between the districts is explained by differing amounts of wood consumption and charcoal production, since the biomass's carbon content is directly related to its mass. On the other hand, the average amount of original carbon in the wood transferred into by-products (the sum of carbon in brands, condensable and ash) were about 91 kg C in the study area. During charcoal production process, not only is all the carbon in the wood transferred into solid and liquid products, but a substantial portion is also released into the atmosphere as gaseous products. Table 4 shows the distribution of the original carbon among solid, liquid, and gaseous products of the charcoal production processes.

**Table 4:** Average carbon content and share of the carbon distribution in the study area.

District	Initial carbon in wood input used for carbonization		Carbon stored in outputs after carbonization				Carbon lost into atmosphere during carbonization	
			Charcoal		By-products			
	(kg)	(%)	(kg)	(%)	(kg)	(%)	(kg)	(%)
Banja	1035.98	100	311.08	30.11	83.38	8.07	641.52	61.82
Fagita	1762.83	100	434.35	24.91	116.43	6.68	1212.05	68.42
Dangila	842.47	100	267.96	32.82	72.02	8.83	502.49	58.34
Average.	1213.76	100	337.80	29.28	90.61	7.86	785.35	62. 86

According to the result, at the end of the charcoal production process, only 428.41 kg C (337.80 + 90.61) remained stored in the solid and liquid products out of the total initial carbon (1213.76 kg C) that was used in the charcoal production process which was 37.14 % (29.28 % + 7.86 %) of the original carbon in the wood (Table 4). Thus, the remaining initial carbon in the wood (i.e., 785.35 kg C = [1213.76–428.41]) must be lost into the atmosphere as gaseous compounds during the charcoal production processes in the study area (Table 4). This implication is that the average carbon mass loss into the atmosphere as gaseous compounds during charcoal production processes was about 63 % in the study area. The determination of the share of carbon emitted as CO_2_ and non-CO_2_ compounds (CH_4_, CO, TNMHC and TSP) will be discussed in section 3.2.

### 3.2 Carbon balance and emission factors

#### 3.2.1 Carbon balance analysis

As indicated earlier, during the charcoal production process, the carbon in the wood biomass is distributed among the solid, liquid and gaseous products. In this section the carbon balance analysis result that allocates the carbon lost during charcoal production among CO_2_, CH_4_, CO, TNMHC and TSP is presented based on the ERs of CH_4_, CO, TNMHC and TSP relative to CO_2_. The ERs derived from literature values are presented in Table 2 above.

First, the average carbon content of the solid and gas products to produce 1 kg of charcoal and the carbon yield of charcoal (carbon in charcoal/carbon in wood) based on the kiln efficiency results found in this study are presented and discussed. The result in Table 5 shows that the average amount of wood used to produce 1 kg of charcoal was higher in Fagita district (6.64 kg) compared to Banja (5.52 kg) and Dangila (5.11 kg) districts, respectively. Therefore, based on a carbon content of 45.5 % of the wood mass on average a lower C in the wood were used in Dangila district (2324.42 g C) compared to Banja (2510.12 g C) and Fagita (3022.25 g C) districts to produce 1 kg of charcoal (or 747.5 g C of charcoal), respectively (Table 5). These results show that the amount of carbon in the wood used to produce 1 kg of charcoal were inversely related to the conversion efficiency. Thus the carbon yield of charcoal decreases as efficiency of the kiln decreases. Table 5 confirm this, as the average carbon yield of charcoal in Dangila district (32.82 %) surpassed that in Banja (30.11 %) and in Fagita (24.91 %) districts, indicating lower efficiency kilns in Banja and Fagita. In this study, on average from the original carbon in the wood (2618.93 g C) used to produce 1 kg of charcoal, about 1671 g of C was released into the atmosphere as gas per kg of charcoal produced (Table 5). This is an average carbon loss of about 63 % per kg charcoal produced in Awi zone as gaseous products. This result suggests that more than half of the carbon in the initial wood lost into the atmosphere was different gaseous products during charcoal production using traditional kiln.

**Table 5:** Carbon balance of the charcoal production process per 1 kg of charcoal produced in Awi zone.

Balance	unit	Banja	Fagita	Dangila	Average	% distribution of carbon
**Input**						
Wood (dry basis)	kg	5.52	6.64	5.11	5.76	
C wood	g	2510.12	3022.25	2324.42	2618.93	100 %
**Process**						
Kiln efficiency	%	18.32	15.16	19.97	17.82	
**Output**						
Charcoal	kg	1	1	1	1	
C Charcoal	g	747.80	747.80	747.80	747.80	29.28 %

C yield	%	30.11	24.91	32.82	29.28	

C Brands	g	151.23	151.37	151.06	151.55	5.94 %
C Ash	g	2.10	2.10	2.10	2.10	0.08 %
C Condensables	g	47.00	47.00	47.00	47.00	1.84 %

C CO_2_	g	1024.73	1360.61	902.35	1095.90	41.24 %
C CO	g	259.87	345.05	228.84	277.92	10.46 %
C CH_4_	g	73.99	98.24	65.15	79.12	2.98 %
C TNMHC	g	188.14	249.81	165.67	201.21	7.57 %
C TSP	g	15.27	20.27	13.45	16.33	0.61 %

C total Gases	g	1561.99	2073.98	1375.45	1670.47	62.86 %

C Lost	%	61.82	68.41	58.34	62.86	

As we can see from this result and extensively discussed in FAO [2] report, kiln efficiency is the most important factor on climate change effect as it affects the amount of GHGs emissions. Kiln efficiency affects the amount of GHGs emission through its effect on the amount of wood requirement for the production of a given amount of charcoal. Lower efficiency kilns require a larger quantity of wood to produce the same amount of charcoal as higher efficiency kilns. Low efficiency kilns also have a higher carbon loss, both of these factors result in higher GHGs than higher efficiency kilns. The average carbon lost per kg of charcoal produced in Dangila with the highest conversion efficiency records the lowest average carbon loss (1375.45 g C), whereas Fagita district with the lowest conversion efficiency records the highest average carbon loss (2073.98 g C) (Table 5). Consequently, the use of a relatively higher conversion efficiency kilns like in the case of Dangila district compared to Fagita district leads to carbon saving of 698.53 g C per kg charcoal produced.

Now the allocation of the total carbon lost among CO_2_, CH_4_, CO, TNMHC and TSP per 1 kg of charcoal produced are presented and discussed here. The result in Table 5 shows that on average out of the total carbon balance (1671 g C) emitted as gaseous products about 41 % (1096 g C) and 22 % (575 g C) of the original carbon in the wood (2619 g C) per kg of charcoal produced was emitted as CO_2_ and PIC (products of incomplete combustion [CO + CH_4_+TNMHC + TSP]), respectively, while only about 29 % and 8 % of the initial carbon was transferred and stored into charcoal and other non-gas by-products respectively. This implies that even if the charcoal were produced with net zero effect on atmospheric CO_2_ by using fully sustainable wood input such as plantation trees like the practice in the study area (i.e., all CO_2_ recycled) and will be used with absolutely no PIC production during its final end use, the use of traditional kiln in the study area still emits nearly a quarter (22 %) of the carbon available in the initial wood as PIC during its production stage. This result is in line with the findings of the technological process used by traditional charcoal production system which is a significant emitter of PIC by previous studies including Smith et al. [23] and Pennise et al. [24]. The implication is that improving the efficiency of the existing traditional kiln enables us to reduce the emission of PICs.

#### 3.2.2 The emission factors of the kilns in the study area

Emission factor is a parameter that quantifies the magnitude of emissions per unit of an activity produced [32]. In this study emission factor is presented in mass basis (g gas per kg charcoal produced) in order to enable us to make a comparison to similar previous studies on emissions from traditional charcoal production process. From the results presented in Table 5 above, this study converts C CO_2_, C CO, C CH_4_, and C TNMHC (i.e., g C gas per kg charcoal produced) to CO_2_, CO, CH_4_, and TNMHC (i.e., g gas per kg charcoal produced) by multiplying by the ratio of their molecular weights to C, respectively. The results of the average emission factors for GHGs (CO_2_ and CH_4_) and non-GHGs (CO and TNMHC) of the charcoal production in the study area are presented in Table 6.

**Table 6:** Average emission factors for charcoal production in Awi zone.

District	No. of kilns	Average Emission factors g of pollutant per kg of charcoal produced				
		CO_2_	CO	CH_4_	TNMHC	non-CO_2_
Banja	6	3757	606	99	282	987
Fagita	6	4989	805	131	375	1311
Dangila	6	3309	534	87	249	869

Average	18	4018	649	106	302	1056

This study shows that the average emission factors of the 18 traditional kilns in three districts were 4018, 106, 649 and 302 g per kg charcoal produced for CO_2_, CH_4_, CO and TNMHC respectively (Table 6). Similar to the results of the carbon balance for CO_2_ and non-CO_2_, the average emission factors for CO_2_, and non-CO_2_ for Fagita district were higher than the emission factors for Banja and Dangila districts, due to the use of relatively lower conversion efficiency kilns in Fagita ditrict compared to the other two districts. As shown in Table 6, the emissions in Fagita district were higher by 1680 g per kg charcoal produced for CO2 and 442 g per kg charcoal produced for non-CO_2_ compared to the emissions in Dangila district, respectively, while it were 1232 g per kg charcoal produced for CO2 and 324 g per kg charcoal produced for non-CO_2_ compared to Banja districts. Meaning, the CO_2_ and non-CO_2_ emission factors in Fagita district were about 25 % and 34 % higher than the emission factors in Banja and Dangila district, respectively, for the same traditional charcoal kilns. This study compared the estimated emission factors to the results of previous studies for the same traditional earth mound kilns. Table 7 presents a summary of the present and previous studies emission factors.

**Table 7:** Comparisons of current & previous earth mound kiln emission studies.

Study	Country	Effi.	Emission factors, g of pollutant per kg of charcoal produced			
			CO2	CO	CH4	TNMHC
Present study in Banja	Ethiopia	18.32 %	3757	606	99	282
Present study in Fagita	Ethiopia	15.16 %	4989	805	131	375
Present study in Dangila	Ethiopia	19.97 %	3309	534	87	249

Present study average	Ethiopia	17.82 %	4018	649	106	302

Quantis [27]	Tanzania	13.1 %	6204	770	154	319
Brocard et al. [34]	W. Africa	27.6 %	1593	254	39	7.2 (as C)
Smith et al. [23]	Thailand	29.8 %	1140	226	27.7	95.3
Penisse et al. [24]	Kenya	27.5 %	1802	224	45	93
Bertschi et al. [33]	Zambia	27 %	1935	346	47.7	117

FAO [2]	World		966-2125	106–270	0–89	8.5–95.33

As shown in Table 7, the results of the emission factors for charcoal production in the three districts of Awi zone falls outside the FAO [2] reported values for the traditional charcoal production for all gases except for CH4 in Dangila district. The emission factors for charcoal production in this study were considerably higher than the emission factors typically found for the same traditional earth mound kilns in Thailand, Kenya, West Africa and Zambia [23,24,33,34]. The large variation in the emission factor is likely due to differences in the efficiency of the kilns used in the study area and the other studies. The average conversion efficiency of the kilns in the present study (with a range of 15.16 %–19.97 %) were lower than the conversion efficiency of the kilns in the previous studies (which ranges from 27 to 29.8 %) [23,24,33,34]. This lower conversion efficiency in the study area probably results in higher emission factors compared to other study findings. However, the conversion efficiency (13.1 % for the traditional charcoal kilns in Tanzania) reported by Quantis [27] is closer to the conversion efficiency estimated for Fagita district (15.16 %) in this study. Similarly, the emission factor values reported by Quantis [27] are the closest to the emission factors of the kilns used in Fagita district in this study. The finding of this study is consistent with Smith et al. [23] and Penisse et al. [24] who concluded that the value of the emission factors differs depending on the differences in conversion efficiencies of the kilns.

### 3.3 Estimates of GHG emissions and their GWI

This section presents the results of total emission from eighteen charcoal kilns and the implication of these emissions on the climate in terms of GWI in Awi zone. Total emissions of CO_2_, CO, CH_4_, and TNMHC were calculated by multiplying total charcoal production by the corresponding emission factors for the three districts. The results in Table 8 below shows that six kilns in Fagita district emitted more CO_2_, CO, CH_4_, and TNMHC than the same number of kilns emitted in Banja and Dangila districts. The reasons for higher gas emissions recorded per kiln in Fagita district than the other two districts may be due to, first the use of the lowest efficient kilns in Fagita district and second Fagita district produced larger amount of charcoal than the other two districts per kiln. Thus, according to the result in Table 8, the total emissions for each of the four gases in Fagita district were higher by 47 % and 59 % compared to the total emissions in Banja and Dangila districts respectively. In other word, the kilns in Dangila emitted about 78 % of the gases compared to Banja district but only about 41 % compared to the kilns in Fagita district. Similarly, the kilns in Banja district emitted only about half (53 %) of the four gases compared to the same number of kilns emitted in Fagita district.

**Table 8:** Estimated total gas emissions from 18 charcoal kilns.

Measured Parameters		Districts			Total
		Banja	Fagita	Dangila	
**Production Process**	Number of kilns	6	6	6	18
	Total wood used (t)	13.661	23.245	11.109	48.015
	Total charcoal produced (t)	2.496	3.485	2.150	8.131
	Ave. Kiln efficiency (%)	18.32	15.16	19.97	17.82
**Gas Emission**	CO2 (t)	9.259	17.492	7.252	34.003
	CO (t)	1.494	2.823	1.170	5.487
	CH4 (t)	0.243	0.459	0.191	0.893
	TNMHC (t)	0.695	1.314	0.545	2.554

This result revealed that, as the amount of charcoal production increases in the case of using relatively less efficient kilns, the total emissions increase at an increasing rate compared to more efficient kilns.

The above result gives the total emissions for the specific gases from the sample charcoal kilns in the study area but does not show their implication on the climate. Thus, in order to see their implication on the climate in terms of GWI, this study converts the total emissions value of the specific gases into reference gas, i.e., CO_2_ as CO_2_-eq based on their GWPs relative to CO2. As previously explained, the charcoal in the study area was produced entirely from a sustainable wood supply, so its production is assumed to be carbon-neutral. However, in this study, we not only calculated the GWI excluding biogenic CO2 but also calculated the GWI including biogenic CO2 emissions. This was done to illustrate the full contribution of CO2 under the assumption that the wood used was entirely sourced from an unsustainable supply.

Table 9 below shows the CO_2_-eq per kg of charcoal produced of the gas emissions and the climate implications of these emissions in terms of PGWI and TGWI for the eighteen kilns by district. This study shows that the main contribution to the GWI corresponds to the emissions of CH_4_ from the charcoal production process in all districts, since this gas has a higher GWP than the other gases (using a 20-year time horizon). This result is consistent with previous studies that show CH_4_ emissions from traditional charcoal kiln was significant [19,21,[23], [24], [25], [26],^31^]. Based on the result in Table 9 the average PGWI (for CH_4_ only in the study area since all CO_2_ is recycled) was 7.6 kg CO_2_-eq per kg of charcoal produced using a 20-year time horizon. The results in Table 9 also shows that, the kilns in Fagita district (with a PGWI of 9.43 kg CO_2_-eq per kg of charcoal produced) contributed 1.3 and 1.5 times more to global warming than the kilns in Banja (with a PGWI of 7.1 kg CO_2_-eq per kg of charcoal produced) and Dangila (with a PGWI of 6.25 kg CO_2_-eq per kg of charcoal produced) districts, respectively. The difference amongst the districts within the study area can be observed, which is mainly explained by the difference in kiln efficiencies.

**Table 9:** Estimated GWI for the 18 charcoal kilns in Awi zone.

		20-year GWP				100-year GWP			
		Banja	Fagit.	Dangil	Ave.	Banja	Fagit	Dangila	Ave.
		(kg CO_2_-eq per kg of charcoal)				(kg CO_2_-eq per kg of charcoal)			
CO_2_		3.76	4.99	3.31	4.02	3.76	4.99	3.31	4.02
CO		2.43	3.22	2.16	2.59	0.79	1.05	0.69	0.84
CH_4_		7.10	9.43	6.25	7.60	2.47	3.27	2.17	2.64
TNMHC		3.39	4.50	2.98	3.62	1.16	1.54	1.02	1.24
all CO_2_ recycled	PGWI	7.10	9.43	6.25	7.60	2.47	3.27	2.17	2.64
	TGWI	12.92	17.15	11.37	13.81	4.41	5.86	3.89	4.72
no CO_2_ recycled	PGWI	10.86	14.42	9.56	11.61	6.22	8.26	5.48	6.66
	TGWI	16.67	22.14	14.68	17.83	8.17	10.85	7.19	8.74

The study findings presented in Table 8, Table 9 implies that attention should be given in improving the efficiency of the traditional charcoal kilns in the study area so as to reduce their relatively high GHG emissions and GWIs. This is more important as the demand of charcoal is expected to be growing and since Awi zone, particularly the Fagita district, serve as the hub of the charcoal industry in the country. Therefore, intervention aiming to improve kiln efficiency can increase the supply of charcoal produced from a given amount of wood and reduce emissions.

However, the result in Table 9 also shows that the charcoal production system in the study area excluding biogenic CO2 emissions has the lowest GWI (7.6 kg CO_2_-eq per kg of charcoal produced) compared to a reference system GWI including biogenic CO2 emissions (11.61 kg CO_2_-eq per kg of charcoal produced). This is attributed to the use of fully sustainably harvested wood in the study area compared to the reference system, which is entirely utilizes unsustainably managed wood sources. This implies that, unlike in the reference system, the production of charcoal using fully sustainable wood reduces the PGWI by about 35 % (4 kg CO_2_-eq per kg of charcoal produced). This finding suggests that the focus should not only be on creating higher efficiency kilns but also on educating charcoal producers on sustainable harvesting techniques.

### 3.4 Impacts of efficiency improvement measures on the reduction of GHG emissions

The study analyzed the impact of improving the conversion efficiency on reducing wood usage and GHG emissions during charcoal production. In charcoal production, simple measures can potentially lead to significant reductions in both wood usage and GHG emissions [2]. One such measure is improving the conversion efficiency of traditional earth mound charcoal kilns, which currently have conversion efficiencies of 10–22 % [2,42]. According to many studies and reports, these traditional kilns have the potential to improve to a minimum threshold of 25 % and up to 35 % conversion efficiency [2,4,42,46]. Based on these reports, this study compares three hypothetical improved kiln technologies with conversion efficiency of 25 %, 30 %, and 35 % against the average efficiency (17.82 %) of the kilns studied. The study compared the environmental impacts using equal amount of charcoal and brand (the average outputs in this study), weighing 451.72 and 131.17 kg, respectively.

The result presented in Table A4 in the appendix revealed that using improved kiln technologies with conversion efficiencies of 25 %, 30 %, and 35 % could have been reduced wood consumption by 28.7 %, 40.6 % and 49.1 %, respectively compared to the average kilns used in the study area to produce the same amount of charcoal. This reduction in wood usage suggests less pressure on natural forests, as charcoal producers can meet their charcoal demand with less wood.

Further, the study also showed that higher kiln efficiency decrease the amount of carbon emitted into atmosphere as CO2 and CH4. For example, using kilns with 25 %, 30 %, and 35 % efficiency could have been reduced the carbon emitted as CO2 by 217.31, 307.20 and 371.41 kg C, respectively, and the amount of carbon emitted as CH4 by 15.69, 22.18 and 26.82 kg C, respectively (Table A4 in the appendix). Similarly, the GWI was also significantly reduced by 45.7 %, 64.6 % and 78.1 % if we use a kiln efficiency of 25, 30 and 35 %, respectively compared to the study area average kiln efficiency (Table A4).

These finding are consistent with other studies, such as those by UNDP [42] and Guidal et al. [6], which highlight that improving kiln efficiency can lead to substantial reductions in GHG emissions and wood consumption. Overall, the study concludes that adopting more efficient kiln technologies in developing countries can play a significant role in mitigating climate change by increasing carbonization efficiency and reducing the volume of wood required for charcoal production.

### 3.5 Study limitations

While offering an overview of the GWI for traditional charcoal production system during the production stage of the charcoal value chain, this study has certain limitations that must be acknowledged when interpreting the results. The conclusions drawn are applicable only within the defined scope, which includes specific geographic regions, system boundaries, and study assumptions. Although the study aims to capture the major aspects of the traditional charcoal production system, particularly focusing on the carbonization stage, not all possible scenarios have been considered.

The analysis of GWI requires an extensive set of data and assumptions, which should be carefully considered when interpreting the results. The carbon balance analysis for emissions estimation relies heavily on literature values due to limited field-measured data for some parameters. The study acknowledges the sensitivity of kiln emissions to local conditions, emphasizing that direct measurement of parameters such as the carbon content of the wood input and outputs (charcoal, brands, and condesables), as well as the ERs in the study area, would enhance data accuracy. Without these direct measurements, the findings may not fully represent the variability and complexity of the real-world conditions.

Furthermore, the study primarily focuses on the GWI, excluding other environmental impacts such as biodiversity, ecosystem services, and human health associated with charcoal production. A comprehensive understanding of the full environmental impacts of traditional earth mound kilns would require a separate, detailed study. This could be accomplished by assembling a team of multidisciplinary experts to investigate each impact concurrently and interconnect the findings across all impacts. In summary, while this study provides valuable insights into the GWI of traditional charcoal production, it is important to interpret the results within the context of its limitations and the assumptions made. Future research with direct measurements and a broader scope of environmental impacts would enhance the understanding and accuracy of the findings.

## 4 Conclusions

The main purpose of the study was to examine the climate impact of charcoal production in Ethiopia with Awi zone as the study area. To achieve these objectives this study collected both primary and secondary data from field measurements on 18 sample kilns in 3 districts of Awi zone and literatures. The results in the study area show that the average carbon loss varies among the 3 districts. It ranges from a lower carbon emission profile per kg of charcoal produced in the best kiln efficiency used in Dangila to a relatively higher emission profile per kg of charcoal produced in the least efficient kiln performance used in Fagita district. Even if the charcoal was produced with sustainable wood source and there is zero net CO_2_ emission, the use of the traditional kiln still emits nearly a quarter (22 %) of the carbon in the original wood as PIC during production process in the study area.

The average emission factors for CO_2_, and non-CO_2_ were higher in Fagita district than in Banja and Dangila districts due to a lower efficiency kiln. Similarly, the kilns in Fagita district with a PGWI of 9.43 kg CO_2_-eq per kg of charcoal produced contributed 1.5 times more to global warming than the kilns in Dangila district with a PGWI of 6.25 kg CO_2_-eq per kg of charcoal produced, which is mainly explained by the difference in kiln efficiencies. However, charcoal production in the study area that uses fully sustainably harvested wood reduces the GWI by about 35 % compared to producing the same amount of charcoal using a production system that utilized entirely unsustainably managed wood sources. In conclusion, the results in this study show that the GHG emissions and GWI of charcoal production using unsustainably managed wood sources and less efficient kiln production systems were higher than in sustainably managed wood sources and more efficient kiln production systems.

Improving the wood sustainability and kiln efficiency components in the traditional charcoal production system is necessary to achieve significant reductions in GHG emissions. This finding is significant to Ethiopia where there is a general lack of quantitative information in informing policy makers the much needed information to achieve the goal of reducing GHG emissions in the charcoal sector prioritized by the Government of Ethiopia. Thus, the policy implications from the findings of this study is that any interventions aiming in mitigating climate change through reduction of GHG emissions from charcoal production must be focused on the combined improvement of the use of sustainably harvested wood input and efficiency of the kiln.

## CRediT authorship contribution statement

**Biruk Belay:** Writing – original draft, Visualization, Methodology, Investigation, Formal analysis, Data curation, Conceptualization. **Dawit Diriba:** Writing – review & editing, Writing – original draft, Supervision, Conceptualization. **Feyera Senbeta:** Writing – review & editing, Writing – original draft, Supervision, Conceptualization.

## Data availability

The data can be accessed upon request from the author.

## Declaration of competing interest

⮽The authors declare that they have no known competing financial interests or personal relationships that could have appeared to influence the work reported in this paper.

## Figures and Tables

**Table A1:** 

Abbreviations and Acronyms	
CDM	Clean Development Mechanism
C	Elemental Carbon
CH4	Methane
CIFOR	Center for International Forestry Research
CO	Carbon Monoxide
CO2	Carbon Dioxide
CO2-eq	Carbon Dioxide equivalent
CRGE	Climate Resilient Green Economy Strategy
EFs	Emission Factors
ERs	Emission Ratios
FAO	Food and Agricultural Organization
g	gram
GHG	Greenhouse Gas
GWI	Global Warming Impact
GWP	Global Warming Potential
IPCC	Intergovernmental Panel on Climate Change
kg	Kilogram
MC	Moisture content
Mt	Million ton
NDC	Nationally Determined Contribution
PGWI	Primary Global Warming Impact
PIC	Products of Incomplete Combustion
SSA	Sub-Saharan Africa
t	ton
TGWI	Total Global Warming Impact
TNMHC	Total Nonmethane Hydrocarbons
TSP	Total Suspended Particulates
UNEP	United Nations Environment Programme

**Table A2:** Conversion factors of non-CO_2_ gases to CO_2_-eq for 20 & 100 year GWP

non-CO_2_ gases	20 year GWPs	100 year GWPs	Source
CH_4_	1gCH_4_ = 72gCO_2_-eq	1gCH_4_ = 25gCO_2_-eq	IPCC [47]
CO	1gCO = 4gCO_2_-eq	1gCO = 1.3gCO_2_-eq	Pennise et al. [24]; Bailis et al. [19]
TNMHC	1gTNMHC = 12gCO_2_-eq	1gTNMHC = 4.1gCO_2_-eq	Pennise et al. [24]; Bailis et al. [19]

**Table A3:** Field measurement results from the 18 sample earth mound kilns in 2020

District	Kiln ID no.	Wood Species used	Kiln Vol. (m3)	Wet wood cut (kg)	Oven dry wood (kg)	MC oven dry basis (%)	Charcoal produced (kg)	Eff. on dry basis (%)
Banja	1	Eucalyptus g.	8	3200	2215	44.47	420	18.96
Banja	2	Accacia d.	6	3105	2341	32.64	350	14.95
Banja	3	Accacia d.	4	2954	2261	30.65	436	19.28
Banja	4	Mix	7	4370	3190	36.99	595	18.65
Banja	5	Eucalyptus g.	4	2590	1848	40.15	320	17.32
Banja	6	Accacia d.	5	2354	1806	30.34	375	20.76
Fagita L.	7	Accacia d.	11	6543	4948	32.24	675	13.64
Fagita L.	8	Accacia d.	7	4132	2880	43.47	495	17.19
Fagita L.	9	Accacia d.	10	5673	4076	39.18	585	14.35
Fagita L.	10	Accacia d.	9	4879	3616	34.93	510	14.10
Fagita L.	11	Mix	8	5468	4154	31.63	640	15.41
Fagita L.	12	Accacia d.	6	4780	3571	33.86	580	16.24
Dangila	13	Mix	3	1039	783	32.69	200	25.54
Dangila	14	Accacia d.	5	3200	2320	37.93	450	19.40
Dangila	15	Eucalyptus g.	6	2950	2129	38.56	465	21.84
Dangila	16	Mix	4	2360	1696	39.15	320	18.87
Dangila	17	Eucalyptus g.	5	2645	1947	35.85	330	16.95
Dangila	18	Eucalyptus g.	5	2987	2234	33.71	385	17.23

**Table A4:** Comparison of the traditional kiln used in the study with 3 improved kilns efficiency

Balance		unit	Efficiency category			
			Study area Efficiency	Efficiency 1	Efficiency 2	Efficiency 3
**Output**						
Charcoal		kg	451.72	451.72	451.72	451.72
Kiln efficiency		%	17.82	25	30	35
**Required input**						
Wood (oven dry basis)		kg	2534.92	1806.89	1505.74	1290.64
C Wood (45.5 % of oven dry wood)		kg	1153.39	822.13	685.11	587.24
Distribution of initial carbon (Carbon basis)	**C in solid and liquid products**					
	C charcoal	kg	337.80	337.80	337.80	337.80
	C brand	kg	68.43	68.43	68.43	68.43
	C condensable	kg	21.23	21.23	21.23	21.23
	C ash	kg	0.95	0.95	0.95	0.95
	**C emit as Gas**					
	C CO2	kg	475.61	258.30	168.41	104.20
	C CH4	kg	34.34	18.65	12.16	7.52
	C CO	kg	120.62	65.51	42.71	26.43
	C TNMHC	kg	87.32	47.42	30.92	19.13
Mass basis	CO2	kg	1743.93	947.11	617.50	382.07
	CH4	kg	45.87	24.87	16.21	10.03
	CO	kg	281.43	152.84	99.65	61.66
	TNMHC	kg	130.98	71.14	46.38	28.70
20-year GWP (total)	CO2	kg	1743.93	947.11	617.50	382.07
	CH4 (CO2-eq)	kg	3296.49	1790.28	1167.24	722.20
	CO (CO2-eq)	kg	1125.73	611.37	398.60	246.63
	TNMHC (CO2-eq)	kg	1571.81	853.63	556.55	344.36
20-yr GWP per kg charcoal	CO2	kg/kg	3.86	2.10	1.37	0.85
	CH4 (CO2-eq)	kg/kg	7.29	3.96	2.58	1.60
	CO (CO2-eq)	kg/kg	2.49	1.35	0.88	0.55
	TNMHC (CO2-eq)	kg/kg	3.48	1.89	1.23	0.76
GWI(all CO2 recycled)	PGWI	kg/kg	7.30	3.96	2.58	1.60
	TGWI	kg/kg	13.27	7.21	4.70	2.91
GWI (no CO2 recycled)	PGWI	kg/kg	11.16	6.06	3.95	2.45
	TGWI	kg/kg	17.13	9.30	6.07	3.75
**GWI reduction Potential of the 3 kilns compared to the study area efficiency**		%	–	45.7	64.6	78.1
